# Growth and Differentiation of Circulating Stem Cells After Extensive *Ex Vivo* Expansion

**DOI:** 10.1007/s13770-021-00330-7

**Published:** 2021-02-24

**Authors:** Silvia Barbon, Senthilkumar Rajendran, Thomas Bertalot, Monica Piccione, Marco Gasparella, Pier Paolo Parnigotto, Rosa Di Liddo, Maria Teresa Conconi

**Affiliations:** 1 Department of Neurosciences, Section of Human Anatomy, University of Padova, Via A. Gabelli 65, 35121 Padova, Italy; 2 Foundation for Biology and Regenerative Medicine, Tissue Engineering and Signaling (T.E.S.) Onlus, 35030 Padova, Italy; 3 Department of Pharmaceutical and Pharmacological Sciences, University of Padova, Via F. Marzolo 5, 35128 Padova, Italy; 4 Department of Woman and Child Health, University of Padova, Via N. Giustiniani 3, 35128 Padua, Italy

**Keywords:** Circulating stem cells, Neurogenesis, Myogenesis, Degenerative diseases, Regenerative medicine

## Abstract

**Background:**

Stem cell therapy is gaining momentum as an effective treatment strategy for degenerative diseases. Adult stem cells isolated from various sources (i.e., cord blood, bone marrow, adipose tissue) are being considered as a realistic option due to their well-documented therapeutic potentials. Our previous studies standardized a method to isolate circulating multipotent cells (CMCs) that are able to sustain long term *in vitro* culture and differentiate towards mesodermal lineages.

**Methods:**

In this work, long-term cultures of CMCs were stimulated to study *in vitro* neuronal and myogenic differentiation. After induction, cells were analysed at different time points. Morphological studies were performed by scanning electron microscopy and specific neuronal and myogenic marker expression were evaluated using RT-PCR, flow cytometry and western blot. For myogenic plasticity study, CMCs were transplanted into *in vivo* model of chemically-induced muscle damage.

**Results:**

After neurogenic induction, CMCs showed characteristic dendrite-like morphology and expressed specific neuronal markers both at mRNA and protein level. The calcium flux activity of CMCs under stimulation with potassium chloride and the secretion of noradrenalin confirmed their ability to acquire a functional phenotype. In parallel, the myogenic potential of CMCs was confirmed by their ability to form syncytium-like structures *in vitro* and express myogenic markers both at early and late phases of differentiation. Interestingly, in a rat model of bupivacaine-induced muscle damage, CMCs integrated within the host tissue taking part in tissue repair.

**Conclusion:**

Overall, collected data demonstrated long-term cultured CMCs retain proliferative and differentiative potentials suggesting to be a good candidate for cell therapy.

## Introduction

Degenerative diseases remain poorly understood and often not curable pathologies associated to heavy toll in disability, death, and far-reaching socioeconomic impacts [1]. Much of biomedical research focuses on discovering the molecular basis of degenerative diseases to define and understand their natural history, etiology and pathogenesis. Thanks to that, modern medicine is accumulating an ever-increasing arsenal of molecular-based diagnostics and therapeutics for a large number of these disorders [2]. Despite increasing knowledge on this field, degenerative disorder treatment still has to face some specific clinical challenges, which include: improving diagnostic capability, particularly early diagnosis, understanding more about mechanisms of disease and developing new therapies aimed at slowing progression or preventing these disorders.

Neurodegenerative diseases result from the gradual and progressive loss of neural cells, leading to nervous system dysfunction [3]. The hallmark of several degenerative disorders in the central nervous system (i.e., amyotrophic lateral sclerosis, Parkinson’s disease, multiple sclerosis, and Alzheimer’s disease) is the massive loss of one or several types of neurons. Although neurological disorders manifest with different clinical features, the disease processes at the cellular level appear to be similar. Cell death and deposition of abnormal proteins and plaques, for example, is a feature common to most neurodegenerative disorders [4]. The development of new therapeutic strategies was once complicated by the fact that the nerve path was first thought to be static, immobile, and incapable of regeneration. In the last decades, much evidence demonstrates that generation of new neurons, namely neurogenesis, is not entirely restricted to prenatal development, but continues throughout adult life in certain regions of the mammalian brain [5]. This may open new perspectives in clinical research and therapy of degenerative neural disorders.

Another class of degenerative disorders regards muscular degeneration, which is a condition marked by the progressive deterioration of muscle tissue, leading to weakness and impaired normal function. Usually, degenerative muscle diseases are marked by problems with walking, balance, and coordination, and many affect speech, swallowing, and even breathing. Some examples of diseases that cause muscle deterioration include muscular dystrophy, which is inherited, and amyotrophic lateral sclerosis, which eventually causes the death of the patient [6]. Current treatment options for degenerative muscular disorders are disappointingly limited and focus mainly on managing symptoms and suppressing the immune and inflammatory response. Therapeutic approaches that aim instead to cure these diseases have been a subject of research for many decades and can be grouped broadly into two categories: the repair or replacement of the mutated gene, and the activation of alternative pathways to reduce the impact of the mutation and correct the pathological consequences [7].

In regenerative medicine, the chronic shortage of organ transplants in conjunction with the limitation of artificial implants (prostheses) has intensified research in cell and tissue-based therapies. The key advantage of cell and tissue therapy over pharmacological therapies to treat debilitating diseases and abnormalities is that the former offers “living biological replacements” while the latter merely provides a palliative solution [8]. Adult multipotent stem cells from peripheral blood present a viable option for easy manipulation to control self-renewal and to direct towards a tissue specific differentiation [9–11].

In this work, human circulating multipotent cells (CMCs) were isolated by a previously reported protocol [10, 11] and characterized for a unique immunophenotype, tested for stability in long-term culture and plasticity towards mesenchymal lineages, in agreement with the guidelines defined by the International Society for Cellular Therapy [12]. In order to investigate their potential use in degenerative neural and muscle disorder treatment, lineage switching capacity of CMCs was verified. Cells were specifically differentiated towards neuronal and skeletal muscle lineages and evaluated for their ability to acquire a functional neuronal phenotype and to integrate in a foreign milieu by using an *in vivo* model of skeletal muscle damage.

## Materials and methods

### Expansive culture and immunophenotypic characterization of CMCs

Before testing shift lineage responses of multipotent cells from peripheral blood, a detailed characterization of cell populations of interest was performed to assess their distinctive stem properties. As previously described by Scapin and co-workers [10], multipotent cells were isolated from human peripheral blood using Ficoll-Histopaque (Merck, Kenilworth, NJ, USA) for density gradient separation. The mononuclear cell fraction was collected, resuspended in stem cell proliferation medium, and seeded onto culture tissue plates, allowing isolation of mesenchymal-like stem cells by plastic adhesion. Adherent cell cultures were stabilized and finally characterized at three different generations (VII, XV, XXXI). First of all, morphologic analysis was performed, using both optical and scanning electron microscopy, and cell proliferation rate was defined by investigating doubling time and population doubling level [11]. Immunophenotype and genotype characterization was performed as described in paragraphs 2.2 and 2.3, respectively. Differentiation experiments towards the three mesodermal lineages (i.e., adipogenic, osteogenic and chondrogenic lineages) were also performed.

### Karyotype analysis

The karyotype study was repeated on three different populations and karyotypic stability was assessed on three different generations. Cytogenetic analysis was carried out by setting up two independent cultures, each obtained by seeding 10^5^ cells on a slide (amnio-dish) placed on the bottom of Petri dishes (Corning, NY, USA). Cells were seeded at a density of 1.2 × 10^3^ cell/cm^2^ in proliferative medium. In the first culture, cells were blocked with colchicine after 24–96 h from seeding, at a confluence of 20%, while the second culture was blocked 24 h after. Subsequently, the metaphases were blocked with colcemid (Merck) and cells were maintained in culture for 12–16 h. The osmotic treatment was then carried out by incubation in hypotonic solution (KCl 0.075 M, Merck) and fixation with a solution of methanol, ethanol and acetic acid (2:1:1, v/v) (Merck). The slides were then extracted from the Petri dishes and dried under controlled temperature (26 °C) and humidity (36% RH) conditions. For the Q-banding method using quinacrine (QFQ), the slides were immersed in a quinacrine (Merck) solution in McIlvaine buffer, kept in the dark for 10 min and, after rinsing in the same buffer, mounted. The metaphases were visualized by fluorescence optical microscopy and acquired through an image analysis system.

### *In vitro* commitment of long-term cultured CMCs

#### Neuronal commitment

CMCs (10^4^ cells/cm^2^) derived from VII, XV, and XXXI subcultures were seeded on 6-well multiwell plates (Corning) previously conditioned with 2% gelatin (Merck) aqueous solution by a 2 h-incubation period at 37 °C. Cells were cultured with inductive medium composed of Neurobasal Medium (Life Technologies, Carlsbad, CA, USA), 2% B-27™ Supplement (Life Technologies), 20 ng/mL Epidermal Growth Factor (EGF) (Merck), 10 ng/mL basic Fibroblast Growth Factor (bFGF) (Merck), 1% glutamax (Merck), and 1% penicillin and streptomycin solution (Merck).

At 3 days from seeding, 50% of medium was replaced with fresh one, whereas, starting from 7 days, cells were cultured for 7 days with inductive medium composed of Neurobasal medium, 2% B-27™ Supplement, 0.5 mM retinoic acid (RA) (Merck), 20 ng/mL Nerve Growth Factor (NGF) (Merck), 1% glutamax, and 1% penicillin/streptomycin solution. Cultures grown in basal medium (Neurobasal Medium, 2% B-27™ Supplement, 1% glutamax, and 1% penicillin/streptomycin) were taken as control.

#### Myogenic commitment

CMCs (10^4^ cells/cm^2^) derived from VII, XV, and XXXI subcultures were seeded on 6-well multiwell plates (Corning) and cultured with proliferation medium composed of Alfa-Modified Eagle Medium (α-MEM) Without Nucleosides (Life Technologies), 16.5% fetal bovine serum (FBS) (Life Technologies), 1% glutamax, and 1% penicillin/streptomycin. On reaching a confluence of 90%, myogenic differentiation was performed using a myogenic induction medium, composed of proliferation medium supplemented with 100 ng/mL insulin-like growth factor I (IGF-I) (Merck) and 200 µM vitamin C (Merck). The medium was changed every 3 days. Cultures grown in proliferation medium were taken as control.

#### Assessment of neurogenic and myogenic differentiative response

CMCs were analyzed at 7 (T7) and 14 (T14) days for neurogenic differentiation response and at 3 (T3), 7 (T7) and 14 (T14) days for myogenic differentiation. At each time point, differentiated cells and corresponding undifferentiated controls (i.e., C3, C7, C14) were investigated by scanning electron microscopy (SEM), flow cytometry, RT-PCR, immunofluorescence, and Western blotting.

- **A) Scanning electron microscopy (SEM)**
  Cultures were fixed with 0.1 M cacodylate buffer solution, pH 7.2 (Merck) in 3% glutaraldehyde (Merck) and dehydrated through increasing concentrations of ethyl alcohol (from 70 to 100%) (Carlo Erba, Milan, Italy). After Critical Point Drying and gold sputtering, images were acquired using Jeol JSM 6490 scanning electron microscope.
- **B) Flow cytometry analysis**
  The immunophenotype characterization of CMCs during the expansion culture and after neurogenic/myogenic induction was carried out by flow cytometry. Cells were detached from the culture dish by EDTA/trypsin treatment, centrifuged at 1200 rpm for 5 min and finally resuspended in phosphate buffer saline (PBS) (Merck), 0.2% bovine serum albumin (BSA) (Merck) (PBS-BSA). Each sample was prepared by treating 2 × 10^5^ cells/100μL PBS-BSA with 5 μl fluorochrome-conjugated or unconjugated primary monoclonal antibodies (Table 1) in the dark for 15 min at room temperature (RT).
  After incubation with the unconjugated primary antibody, samples were rinsed in PBS-BSA and treated with the PE-conjugated secondary antibody in the dark for 15 min at RT. In parallel, control cells were labeled with the secondary antibody or the isotype control (Table 1). Then, all samples were rinsed with PBS-BSA, centrifuged, and resuspended samples in 200 μl of FacsFlow buffer (BD Biosciences, Franklin Lakes, NJ, USA). Analysis was carried out by using FACS Canto II (BD Biosciences). Data relative to 10^4^ total cells were acquired using FacsDIVA software. Results were expressed as percentage of positive cells compared to the control sample and obtained by the statistical function Substraction of *Summit 4.3* software.
- **C) Gene expression study**
  During the *in vitro* expansion culture, CMCs were tested for gene expression profile of specific markers associated to the self-renewal and pluripotency of stem-like cell populations. First of all, total RNA was extracted from cells by using Trizol® Reagent (Merck) following the manufacturer’s instructions. After spectrophotometric quantification, 1 µg RNA was reverse transcribed into cDNA with reverse transcriptase enzyme and a mixture of hexanucleotides, by using the *Thermoscript™ RT-PCR System* kit (Life Technologies) following the manufacturer’s protocol. After reverse transcription, cDNA was amplified by using the oligonucleotides listed in Table 2 for the identification of pluripotency genes.
  The reaction was carried out with the *Platinum® SYBR® Green qPCR SuperMix-UDG* kit (Life Technologies) and the *DNA Engine Opticon®* Real-Time Thermal Cycler (MJ Research, Waltham, MA, USA). Amplification of cDNA was performed through 40 cycles of denaturation at 95 °C (30 s), annealing at 50 °C (30 s), and primer extension at 72 °C (1 min). Melting curves were obtained in a temperature range from 55 to 95 °C. Data were elaborated by the Opticon Monitor 3 software and results were expressed as ∆Ct, that is the difference between the threshold cycle (Ct) of the calibrator gene (HPRT) and the Ct of the target gene.
  In parallel, the acquired expression of specific lineage genes by differentiated CMCs was assessed by semi-quantitative One Step RT-PCR. After total RNA was isolated and quantified as described above, the Qiagen® One Step RT-PCR Kit (Qiagen, Hilden, Germany) and the thermal cycler iCycler iQ™ (Bio-Rad, Hercules, CA, USA) were used to carry out in a single tube the reverse transcription of RNA into cDNA and its amplification. Following the manufacturer’s protocol, RNA samples (30 ng/tube) were amplified to assess the expression of the neuronal and muscle markers listed in Table 3.
  The electrophoretic analysis of PCR reaction products was performed by running amplicons (6 μL) on 2% agarose gel (Merck) and using a reference marker for molecular weights between 100 and 1000 base pairs. The bands of amplified samples were visualized by GelRed® Nucleic Acid Gel Stain (0.1μL/ml) (Biotium, Fremont, CA, USA) staining and exposure to UV light. Images were acquired through Gel Doc 2000 (Bio-Rad) and band densities were quantified with ImageJ software.
- D) Protein expression study

**Table 1:** Antibodies tested for flow cytometry analysis on undifferentiated and differentiated CMC. PE: Phycoerythrin; PE-Cy7: Phycoerythrin-cyanine7

Antibodies	Manufacturing company
**Primary antibodies**	
*Stem cell markers*	
PE mouse anti-human CD14	Santa Cruz Biotechnology
PE mouse anti-human CD45	Santa Cruz Biotechnology
PE-Cy7 mouse anti-human CD34	BD Biosciences
mouse anti-human CD13	BD Biosciences
mouse anti-human NG2	Santa Cruz Biotechnology
PE mouse anti-human CD73	BD Biosciences
PE mouse anti-human CD90	Santa Cruz Biotechnology
PE mouse anti-human CD105	Santa Cruz Biotechnology
PE mouse anti-human HLA-DR	Santa Cruz Biotechnology
*Neurogenic markers*	
mouse anti-human Tyrosine Hydroxylase (TH)	Merck Millipore
mouse anti-human Microtubule Associated Protein 2 (MAP2)	Merck Millipore
mouse anti-human βIII Tubulin (TBB3)	Merck Millipore
*Myogenic markers*	
mouse anti-human Tropomyosin (TPM)	Santa Cruz Biotechnology
mouse anti-human Myogenic factor 5 (Myf5)	Santa Cruz Biotechnology
mouse anti-human Myoblast determination protein 1 (MYOD)	Santa Cruz Biotechnology
**Secondary antibodies**	
PE goat anti-mouse	Santa Cruz Biotechnology
**Isotype controls**	
PE Isotype Control	Santa Cruz Biotechnology
PE Isotype Control	BD Biosciences
PE-Cy7 Isotype Control	BD Biosciences

**Table 2:** Primers for Real-Time RT-PCR

Primers	Abbreviation	Sequence (5′-3′)	Reference	Lengh
Nanog homeobox	NANOG	F-CGGACAAAGAGTTGGCTGTGCAAT R-AGCTGGGTGGAAGAGAACACAGTT	NM_024865.2	106 pb
POU class 5 homeobox	OCT4	F-TATGCAAAGCAGAAACCCTCGTGC R-TTCGGGCACTGCAGGAACAAATTC	NM_002701.4	102 pb
Zinc finger protein, omolog 42	REX1	F-TGGAGGAATACCTGGCATTGACCT R-AGCGATTGCGCTCAGACTGTCATA	NM_174900.3	105 pb
Sex determining region Y box 2	SOX2	F-CACATGAAGGAGCACCCGGATTAT R-GTTCATGTGCGCGTAACTGTCCAT	NM_003106.3	191 pb
Kruppel-like factor 4	KLF4	F-TGAACTGACCAGGCACTACCGTAA R-TCTTCATGTGTAAGGCGAGGTGGT	NM_004235.4	106 pb
Gene coding for p67 myc protein	c-Myc	F-ACAGCATACATCCTGTCCGTCCAA R-TGTTCTCGTCGTTTCCGCAACAAG	D10493.1	79 pb
Signal transducer and activator of transcription 3	STAT3	F-ATGGAAGAATCCAACAACGGCAGC R-GGTCAATCTTGAGGCCTTGGTGA	NM_213662.1	175 pb
Hypoxanthine phosphoribosyltransferase 1	HPRT1	F-ATGGACAGGACTGAACGTCTTGCT R-TTGAGCACACAGAGGGCTACAATG	NM_000194.2	79 pb

**Table 3:** Primer pairs for RT-PCR study

Primers	Abbreviation	Sequence (5′-3′)	Reference	Length
Secreted Protein Acidic and Cysteine Rich	SPARC	F-TCAAGAACGTCCTGGTCACCTTGT R-ATCCTTGTCGATGTCCTGCTCCTT	NM_001031794.1	388 pb
Microtubule-associated protein 2	MAP2	F-GAGGTTGCCAGGAGGAAATCAGT R-GCCCTGAAGCCATCTGTCCAAA	NM_002374.3	703 bp
Synaptophysin	SYP	F-TGTGAAGGTGCTGCAATGGGTC R-GGGCCCTTTGTTATTCTCTCGGT	NM_003179.2	337 bp
Glutamate-Aspartate transporter	GLAST	F-ATCGCCTGCCTGATCTGTGGAAA R-AACGAAAGGTGACAGGCAAAGT	U01824.1	249 bp
Neurofilament, medium polypeptide	NEFM	F-AATATGCACCAGGCCGAAGAGT R-AAATGACGAGCCATTTCCCACT	NM_005382.2	296 bp
Neurogenin 1	NEUROG1	F-GCGCTTCGCCTACAACTACATCT R-TGAAACAGGGCGTTGTGTGGAG	NM_006161.2	301 bp
Nestin	NES	F-GACACCTGTGCCAGCCTTTCTTA R-TGCTGCAAGCTGCTTACCACTTT	NM_006617.1	469 bp
βIII tubulin	TBB3	F-CAACGAGGCCTCTTCTCACAAGT R-TACTCCTCACGCACCTTGCTGAT	NM_006086.3	325 bp
Nerve Growth Factor	NGF	F-GCCCACTGGACTAAACTTCAGCA R-GATGTCTGTGGCGGTGGTCTTA	NM_002506.2	356 bp
Brain-derived neurotrophic factor	BDNF	F-GCAAACATCCGAGGACAAGGTG R-GCTCCAAAGGCACTTGACTACT	NM_170735.5	244 bp
Glial-derived neurotrophic factor	GDNF	F-GCGCTGAGCAGTGACTCAAATA R-GTTTCATAGCCCAGACCCAAGT	NM_000514.3	275 bp
Neuronal Nuclei Antigen	NeuN	F-ACCAACGGCTGGAAGCTAAATC R-ATCCATCCTGATACACGACCGCT	NM_001082575.1	216 bp
Myogenic factor 5	Myf5	F-ACCCTCAAGAGGTGTACCACGA R-ACAGGACTGTTACATTCGGGCA	NM_005593.2	213 bp
Myoblast determination protein 1	MYOD1	F-GCCACAACGGACGACTTCTATGA R-GGCCTCATTTACTTTGCTCAGGC	NM_002478.4	316 bp
Tropomyosin	TPM	F-AGCACATTGCTGAAGATGCCGAC R-AGCTTGTCGGAAAGGACCTTGA	NM_001018005.1	244 bp
Skeletal α-actin	ACTA1	F-TCACGAGACCACCTACAACAGCA R-CTCCTGCTTGGTGATCCACATCT	NM_001100.3	263 bp
Glyceraldehyde 3-phosphate dehydrogenase	GAPDH	F-GGTCGGAGTCAACGGATTTGGT R-AAAGTGGTCGTTGAGGGCAATG	NM_002046.3	887 bp

### Western blotting

After treatment of CMCs with Trizol (Merck), proteins in the phenol-chorloform fraction were extracted according to the manufacturer’s protocol and quantified through the BCA Protein Assay Reagent Kit (Pierce Biotechnology, Waltham, MA, USA).

For the analysis of neurogenic and myogenic markers, 5 μg of total protein were separated using 10% polyacrylamide gel electrophoresis. In parallel, 5 μl ProteinTM, the Precision Plus Dual Color (Bio-Rad) for the definition of the molecular weight reference, were loaded. Electrophoretic run was carried out at 140 V for 2 h. Then, the gel was transferred to polyvinylidene fluoride (PVDF) membranes before performing immunoblotting. To prevent any non-specific interactions with the antibody, membranes were saturated in 5% milk (Merck) in PBS for 2 h and then treated overnight at 4 °C with the following primary antibodies (Merck Millipore): anti-human Tyrosine hydroxylase (TH) and anti-human β-III tubulin (TBB3), both diluted 1:1000 in 1% milk in PBS; anti-human Myogenin (MYOG), anti-human myosin heavy chain (MHC) and anti-human neuron specific enolase (NSE), diluted 1:500 in 1% milk in PBS. Glyceraldehyde 3-phosphate dehydrogenase (GAPDH) antibody (Merck Millipore; diluted 1:1000) was used to detect housekeeping protein. Then, membranes were incubated for 1 h with a solution of the secondary antibody conjugated with horseradish peroxidase (HRP) (Bio-Rad), diluted (1:5000) in 1% milk in PBS. The signal detection was performed through incubation with the Chemiluminescent Peroxidase Substrate (Merck) for 1 min. Once dried, membranes were placed in a Deposit autoradiography, in the dark and in contact with an autoradiographic film (Merck) for a variable time depending on the antibody (5–30 min). The impressed plate was developed by incubation in liquid development XOMAT EX II and fixing RP X-OMAT LO (Kodak, Rochester, NJ, USA). The protein expression level was normalized to housekeeping protein GAPDH and quantified using the image processing software ImageJ.

#### Immunofluorescence

The immunolocalization of the target markers was performed by indirect staining with anti-human primary antibodies against TH, Neuronal Nuclei (NeuN) (Merck Millipore), Neurofilament (NEFM) (Merck Millipore), Dopamine Transporter (DAT) (Santa Cruz Biotechnology, Dallas, TX, USA), Musashi (Merck Millipore), Nestin (NES) (Merck Millipore), Neural Cell Adhesion Molecule (NCAM) (Merck Millipore). For myogenic differentiation, anti-human MYOG and MHC were used. Samples were fixed in BD CytofixTM Fixation Buffer (BD Biosciences) for 20 min at 4 °C, treated overnight at 4 °C with the primary antibody and incubated with FITC-conjugated secondary antibody (Merck Millipore) for 30 min, at RT. Then, samples were mounted with the mounting medium with DAPI (Merk) for nuclear counterstaining.

#### Measurement of calcium flux

This analysis allowed us to determine whether the cells, following neuronal induction, could acquire the ability to transport and release intracellular calcium, a typical feature of excitable cells of the nervous system. Indeed, calcium plays an important role as a mediator in the transduction of transmembrane signals. After 7 and 14 d of induction, the treated samples (T7 and T14, respectively) and controls (C14) were stimulated with 56 mM KCl for 30 min at RT. Each sample was then incubated for 30 min at 37 °C with 5 μl of calcium fluorescent indicator Indo-1 AM (Life technologies), a membrane-permeable dye used for determining changes in intracellular calcium concentrations through fluorescence signal detection. Once Indo-1 enters the cell, esterases cleave the AM group leading to a membrane-impermeable dye. Upon binding calcium, there is a shift in the peak emission of Indo-1. The analysis was carried out in PBS at 37 °C, before and after cell permeabilization with 0.05% Triton X-100 (Merck). Fluorescence (excitation 355 nm, emission 355–600 nm) was read using the Jasco FP-6500 spectrofluorometer.

#### HPLC analysis of neurotransmitter release

At 7 and 14 days of neuronal differentiation, culture media were removed and cells were treated with 56 mM KCl in Hank's Balanced Salt Solution (HBSS) (Thermo Fisher Scientific, Waltham, MA, USA) for 30 min at 37 °C. The supernatant was collected, and separation of the injected samples (20 μL) was achieved by isocratic elution on a Hewlett- Packard Series 1050 HPLC system with a reverse-phase C18 column (3 μm particle size, 80 × 4.6 mm dimension) (ESA, Inc., Chelmsford, MA, USA) in a commercially available MD-TM mobile phase (ESA, Inc.). The flow rate was set at 1 mL/min, resulting in a working pressure of 100 bar and the results were validated by co-elution with noradrenaline standard under varying buffer conditions and detector settings.

### *In vivo* study

Animal procedures were approved by the Ethical Committee of Padua University and by the Italian Department of Health (Project n. CEASA 14 BIS/2011, Protocol n. 26,466, May 5th, 2011). Under isoflurane anesthesia (3% isoflurane carried by oxygen, 1L/min), the *tibialis anterior* muscle of adult Lewis rats (n = 12) was exposed. A narrow gauge (27G) was inserted into each of the ends and in the middle of the muscle, and 0.5 mL of 0.5% (w/v) bupivacaine hydrochloride (Merck) solution were injected [13]. After surgery, the animals were treated with antibiotic (Baytril, 0.2 mL/kg) and analgesic (5 mg/Kg Tramadol, Contramal) for 2 days. CMCs were stained with 15 nM Qdot 800 (cell labeling kit, Life Technologies) for 45 min at 37 °C and then washed with PBS to remove the excess of stain. Forty-eight hours after the bupivacaine injection, 0.5 mL sterile phosphate buffer saline, containing (n = 6) or not (n = 6) 1.5 × 10^5^ pre-labelled CMCs, were injected into the muscle, using the same procedure described above. No immunosuppressive therapy was carried out. After 7 and 14 days, animals were sacrificed by CO_2_ inhalation and their *tibialis anterior* muscle was removed, fixed using isopentane and liquid nitrogen vapors. Cryosections (5–7 µm thickness) were stained as already described at point 2.6.3, using anti-human vimentin antibody (Cell Signaling Technology, Danvers, MA, USA) and mounting medium with DAPI.

### Statistical analysis

Data were expressed as mean ± standard deviation of three different replicates. Statistical analysis was performed by two-way analysis of variance (ANOVA) and Dunnett multiple comparison test, considering *p* ≤ 0.05 to be statistically significant. Statistical calculations were carried out by Prism 8.1.0 (GraphPad Software, San Diego, CA, USA).

## Results

### CMCs long-term cultures retain stemness profile and differentiation potential towards mesodermal lineages

**Fig. 1:**
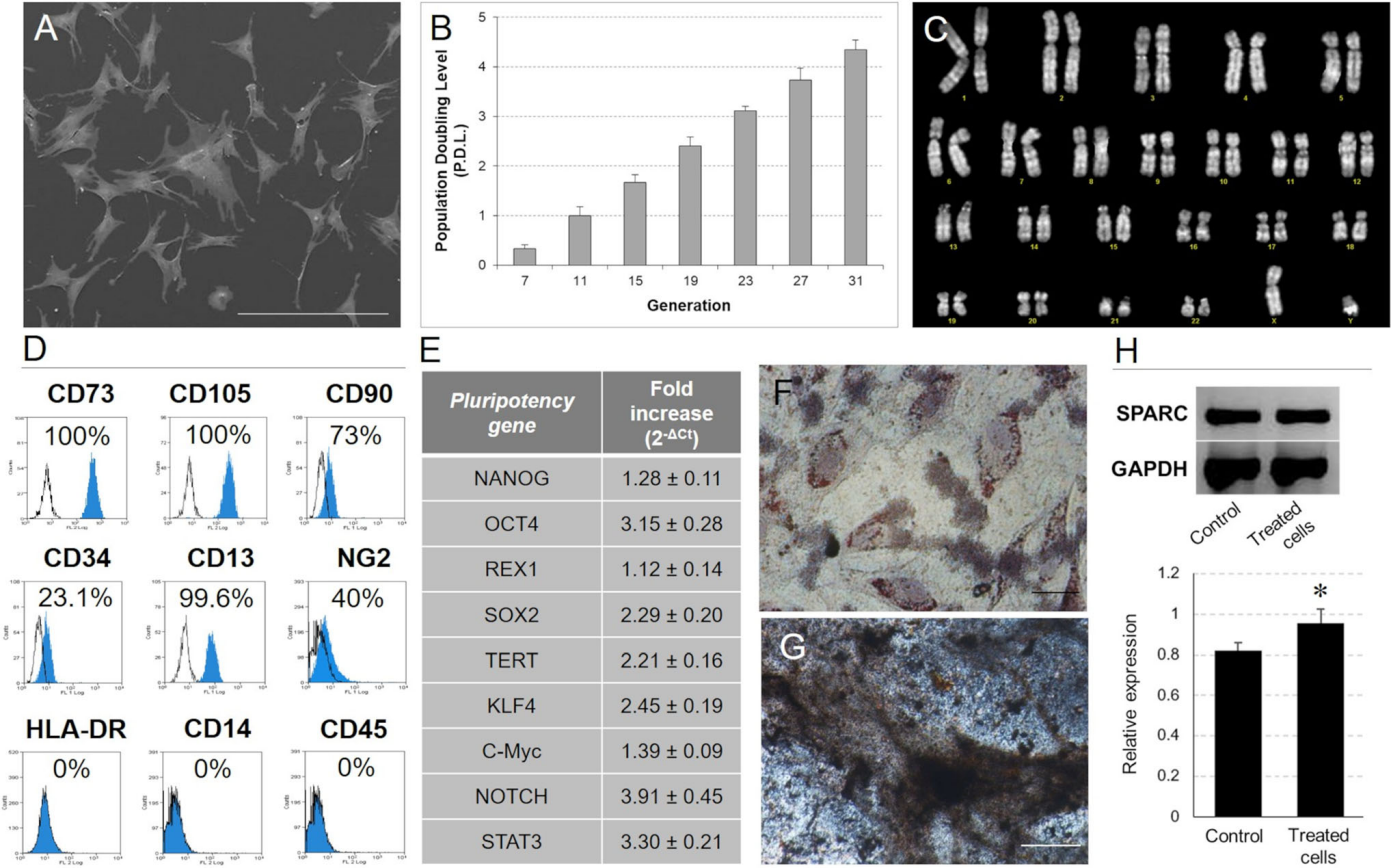
*In vitro* characterization of CMCs. **A** Morphological analysis by SEM. Scale bar: 300 µm. **B** Population doubling study. **C** Karyotype analysis. **D** Immunophenotyping by flow cytometry. **E** Pluripotency gene expression profile identification by qPCR. **F** Adipogenic (Oil Red O) and **G** osteogenic (Von Kossa) differentiation responses. Scale bar: **F** 10 µm; **G** 50 µm. **H** SPARC gene expression detected by RT-PCR (top) and densitometry band quantification (bottom) in CMCs after 20 days of chondrogenic induction (treated cells) in comparison with the control (untreated cells). (*p < 0.05)

In this study, we demonstrated that, after extended *in vitro* culture, CMCs retained their phenotypic features (Fig. 1A), long-term proliferative potential (Fig. 1B) and genetic stability (Fig. 1C). From 2nd to 31st passage, CMC cells showed to express CD105, CD90, CD34, CD73, CD13, NG2 but not hematopoietic markers (CD14, CD45) or HLA-DR (Fig. 1D). Likely Bone Marrow Stem Cells identified as a good candidate for tissue repair strategies [14], CMCs showed the expression of nestin by immunofluorescence. In parallel, the expression of mRNA for several pluripotency genes, such as Nanog, OCT4, Rex1, Sox2, TERT, Klf4, c-Myc, NOTCH and STAT3, confirmed their uncommitted state and multipotency (Fig. 1E). In particular, in accordance with our published data, long-term cultured CMCs were shown to differentiate in vitro acquiring the ability to i) accumulate cytoplasmic lipid droplets, as adipocytes (Fig. 1F); ii) produce mineralized ECM, as osteoblasts (Fig. 1G); and iii) stimulate the gene expression of SPARC, as chondrocytes (Fig. 1H). The responsivity of CMCs to specific lineage inducers suggested that the differentiative potentials were not affected by a prolonged in vitro expansion.

### *In vitro* neurogenic commitment of long-term cultured CMCs

**Fig. 2:**
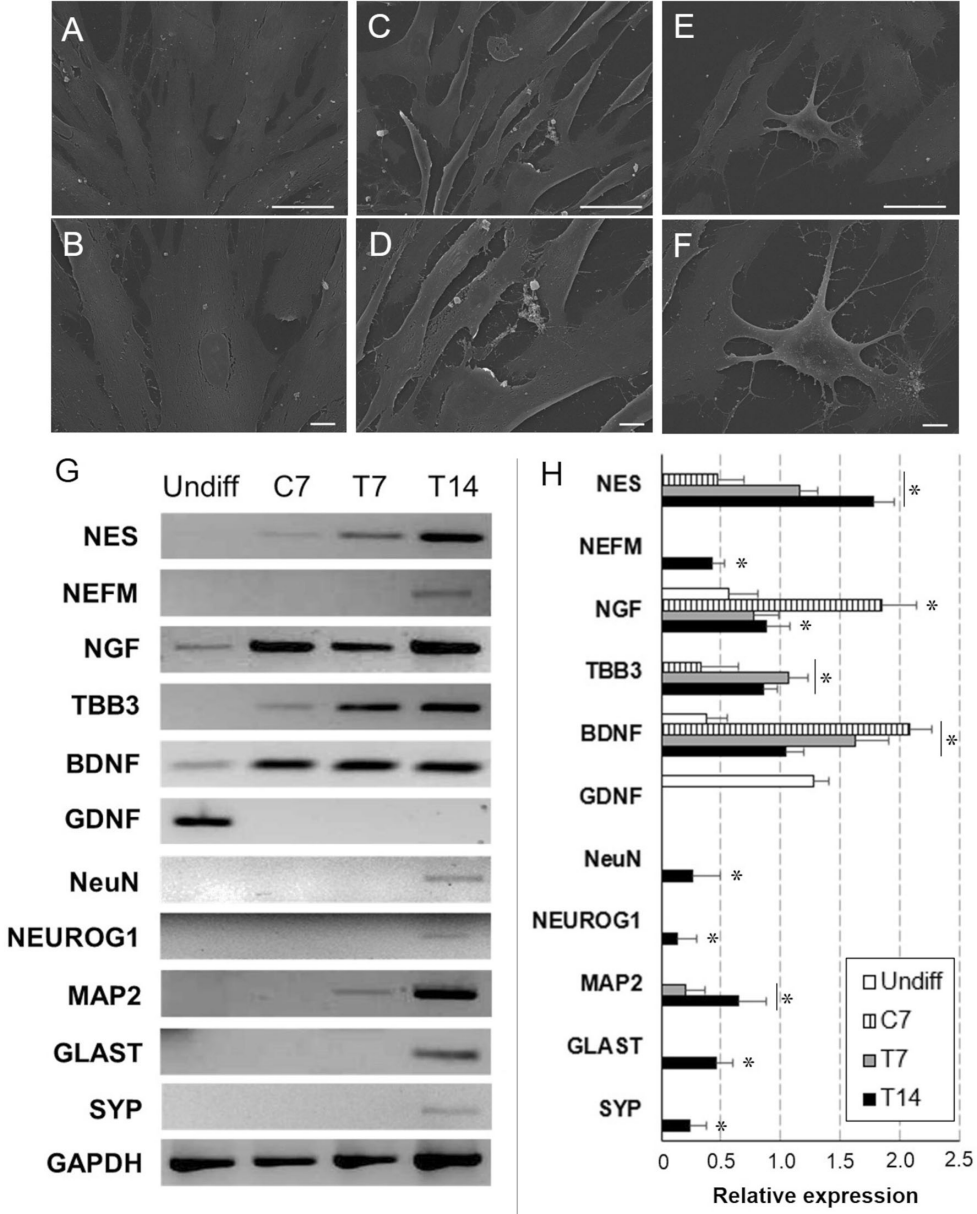
**A–F** Morphological study by SEM of CMCs cultured in NeuroBasal Medium (**A**, **B**), treated with EGF (20 ng/mL) and bFGF (10 ng/mL) for 7 days (**C**, **D**) and then with Retinoid Acid (RA) (0.5 µM) and NGF (20 ng/mL) up to 14 days (**E**, **F**). Scale bar: **A**, **C**, **E** 50 µm; **B**, **D**, **F** 10 µm. **G** OneStep RT-PCR study of neuronal marker expression of CMCs grown in proliferation medium (Undiff), control cells (C7) and cells treated for 7 and 14 days (T7, T14) with inductive factors. In parallel, the expression of the housekeeping gene GAPDH was detected in all samples. **H** Relative quantification of electrophoresis gel bands by densitometric analysis. (*p ≤ 0.05 *vs.* undifferentiated sample)

As reported in Fig. 2A, B, cells grown in Neurobasal medium (*control sample*, C7) preserved the elongated and fibroblast-like morphology (Fig. 2A, B) but gradually detached from culture plates. When Neurobasal medium was supplemented with EGF and bFGF, CMCs responded within 7 days (*treated sample*, T7), undergoing evident changes of morphology and acquiring a spindle shape (Fig. 2C, D). With the additional stimulation of T7 for 7 days with RA and NGF (*treated sample*, T14), CMCs showed a neuron-like morphology, with a polygonal cell body and thin, branched cellular processes (Fig. 2E, F).

In the logarithmic growth phase (*untreated samples)*, CMCs were shown to express genes coding for neurotrophins, such as brain derived neurotrophic factor (BDNF), glial derived neurotrophic factor (GDNF) and NGF (Fig. 2G, H), as already demonstrated for blood-derived mononuclear cells at steady state [15–17].

**Fig. 3:**
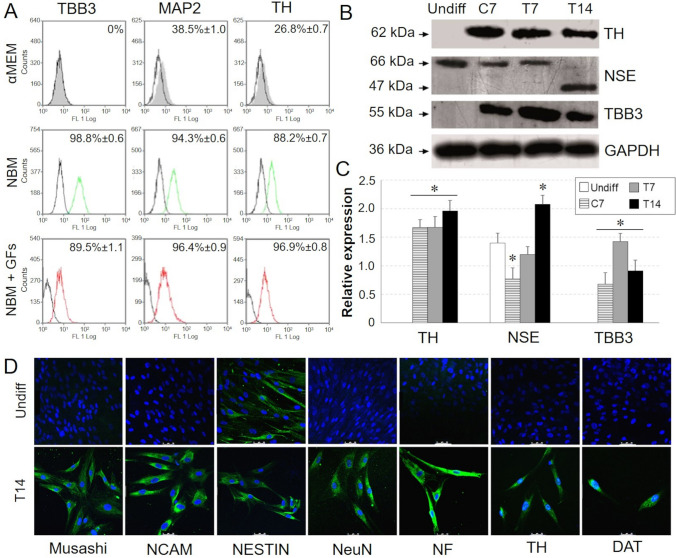
**A** Flow cytometrical analysis of TBB3, MAP2, TH on induced (T14) CMCs (coloured profile) compared to cells cultured in αMEM, NBM, and NBM supplemented with neurogenic factors for 14 days (black profile for all). Indirect staining with FITC-conjugated secondary antibodies was used. For each marker, data were expressed as % positives ± SD of T14 versus C14. **B** Western Blot analysis on undifferentiated (Undiff), C7, T7 and T14 CMCs using 10 g protein extract and chemiluminescence detection. **C** Densitometric quantification of Western Blot bands. (*p ≤ 0.05 *vs* undifferentiated sample). **D** Immunofluorescence of neuronal lineage markers on induced CMCs (T14) compared to undifferentiated samples (Undiff). Cells were indirectly labeled using FITC-conjugated secondary antibodies and images were acquired with the Leica TCS SP5 confocal microscope. Scale bar: 50 µm

The detection of TBB3 mRNA in C7 samples (Fig. 2G, H) and the protein expression of TBB3, MAP2 and TH (Fig. 3A–C) suggested a weak neuronal commitment of CMCs following stimulation with EGF and bFGF included in Neurobasal medium. The gene expression of MAP2, microtubule-associated protein 2, was demonstrated in both T7 and T14, while the expression of late neuronal markers, such as neurofilament medium polypeptide (NEFM), neuronal nuclear antigen (NeuN), Musashi, neural cell adhesion molecule (NCAM) Glutamate-Aspartate transporter (GLAST), synaptophysin (SYP), and neurogenin 1 (NEUROG1), (Fig. 3D) was detected only in T14. Moreover, in T14 cultures the expression of enzymes involved in dopamine biosynthesis, such as TH, and DAT, that is a membrane-spanning protein that pumps dopamine into the synapse cleft, was assumed as the biological marker of acquired neuronal functionality.

The evidence that retinoic acid and nerve growth factor boosted CMCs towards a mature neuronal phenotype was highlighted by the detection of the active form (47 KDa) of NSE in T14 samples (Fig. 3B, C) while in all other samples we detected the inactive form of enolase (66 KDa).

**Fig. 4:**
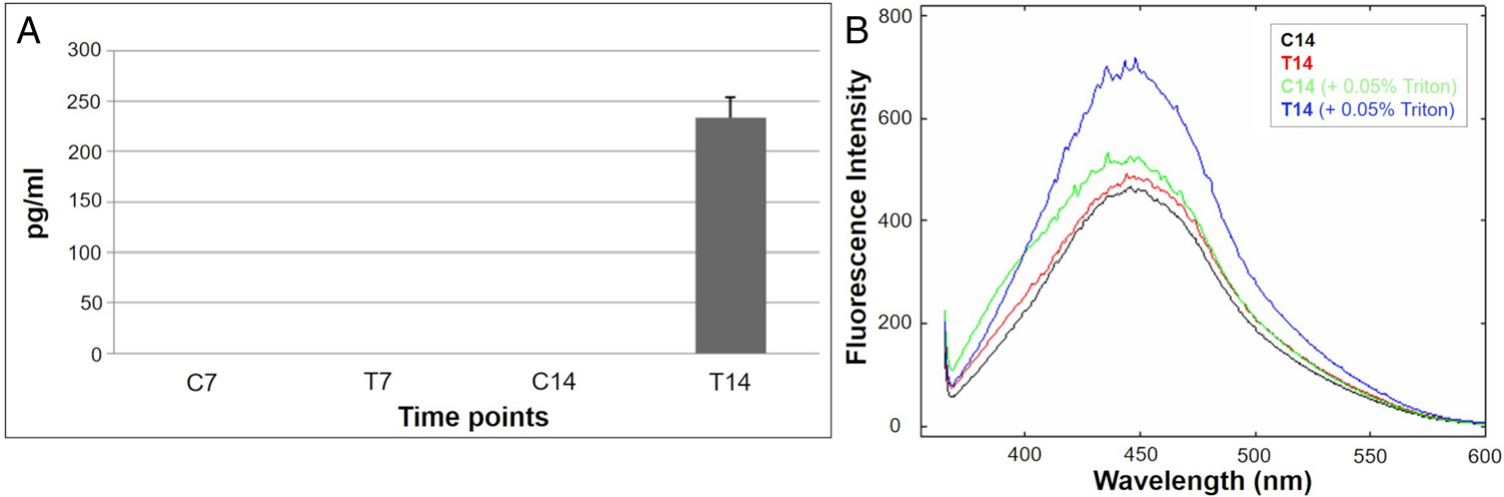
**A** Noradrenaline release by HPLC in CMC cultures after neurogenic induction. **B** Emission spectrum of the fluorescent calcium indicator Indo1 AM (5 μM) after incubation (30 min, 37 °C) with differentiated CMC cultures (C14, T14) previously treated with KCl 56 mM

The progression of CMCs towards neuronal differentiation pathway was also confirmed in T14 cells by the release of noradrenaline (Fig. 4A) and the accumulation of intracellular calcium after KCl stimulation. Calcium flux promotes the synaptic signal transduction and could be measured using Indo-1, a fluorescent calcium indicator. As reported in Fig. 4B, after stimulation with KCl and cellular permeabilization with Triton X-100, both intracellular and extracellular calcium content was demonstrated to be enhanced only in T14 cultures.

### *In vitro* myogenic commitment of long-term cultured CMCs

**Fig. 5:**
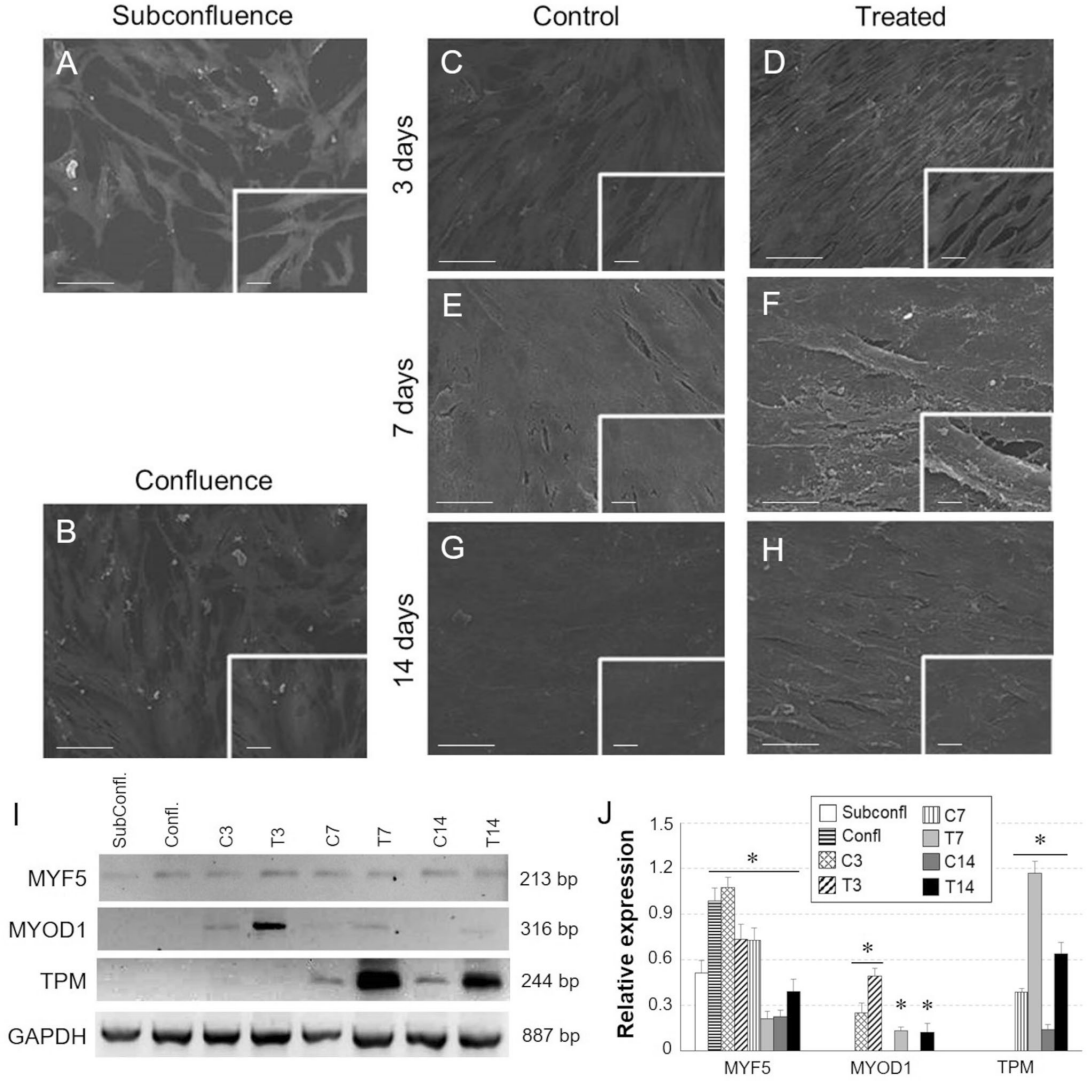
**A–H** Morphological study by SEM of CMC cultures at subconfluent (subconfl) state, at 100% confluence (Confl) and ± treatment with myogenic inductive medium for 3, 7 and 14 days. Scale bar: **A**–**D** 100 µm; **E**–**H** 50 µm. **I** Gene expression analysis by One step RT-PCR of myogenic markers in differentiated CMC cultures. GAPDH was taken as housekeeping gene. **J** Densitometric quantification of Western Blot bands by ImageJ software. (*p ≤ 0.05 *vs.* cultures at subconfluent state)

At 3 days, cultures grown in proliferation medium (*unstimulated sample)* showed a fibroblast-like morphology and a random distribution on the surface plate. At 7 and 14 days, they reached the maximum of confluence and started to stratify. In contrast, under myogenic stimulation, cells aligned parallelly to each other at 3 days, formed packed bundles at 7 days, stratified and fused to each other at 14 days to form multinucleated structures resembling myotubes (Fig. 5A–H).

**Fig. 6:**
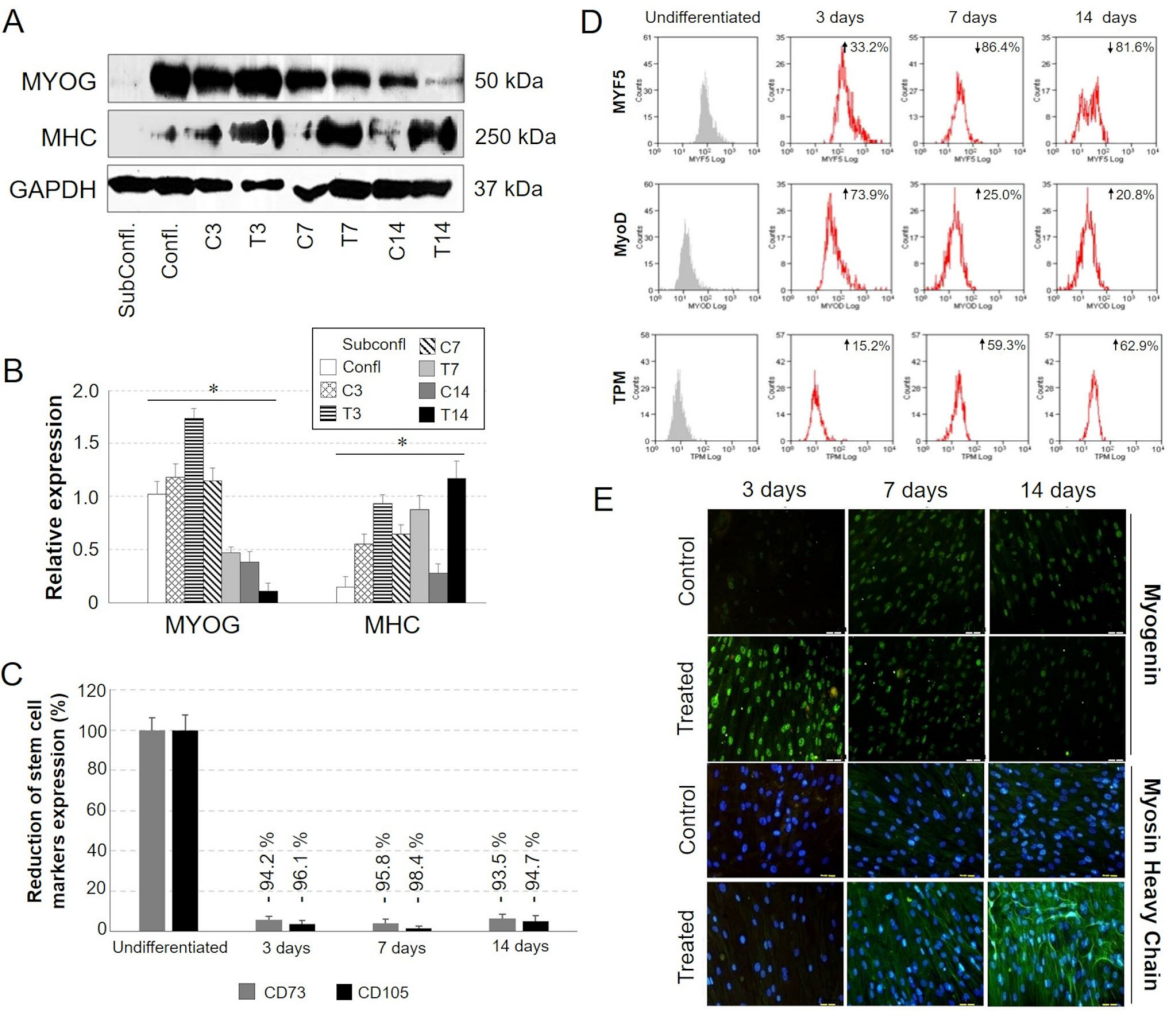
**A**–**E** Protein expression analysis by Western Blot flow cytometry and immunofluorescence of myogenic markers in CMCs differentiated towards myogenic lineage. **B** Quantification of WB bands by densitometry analysis. (*p ≤ 0.05 *vs.* cells at subconfluent state). **C, D** Data are expressed as percentage of positive induced cells compared to undifferentiated control cells. (Subconfl: cultures at a subconfluent state; Confl: cells at 100% confluence). **E** Scale bar: 50 µm

Based on RT-PCR analysis, myogenic medium showed to mimic the *in vivo* myogenic induction, promoting the expression pattern of Myogenic factor 5 (MYF5), Myoblast determination proteins, including MYOD1 and myogenin (MYOG). At 3 days from induction, mRNA and protein expression of both Myoblast determination proteins was significantly higher in samples cultured in differentiative medium, compared to controls (Fig. 5I, J; Fig. 6A, B) but MYF5 and MYOD1 expression was gradually reduced during the differentiation time course (Fig. 6). The temporal expression pattern of MYOG started at 3 days from induction, then was maintained at 7 days and finally faded away at 14 days (Fig. 6A, B, E). The *in vitro* differentiation of CMCs by IGF-I and ascorbic acid seemed to be coherent with the time specific expression pattern of skeletal muscle differentiation markers *in vivo*, showing at early phase the expression of MYF5, followed by MyoD, and MYOG at late phase.

Along with the strong reduction of stem cell marker expression (i.e., CD73, CD105) starting from day 3 after induction (Fig. 6C), the myogenic progression of CMCs was confirmed by the acquired expression of sarcomeric proteins, such as myosin and tropomyosin (TPM). The protein expression of myosin heavy chain (MHC) was not detectable at early phase of induction and started to be evident from 7 days, and gradually increased during the differentiation time course (Fig. 6A, B, E). As shown in Figs. 5I, J and 6D, the expression of sarcomeric tropomyosin started from 3 days of induction, progressively increasing in the last phase of differentiation.

### *In vivo* response of CMCs to a chemically-induced muscle damage

Two days after a standard infiltration of the rat *tibialis anterior muscle* with bupivacaine chloridrate, Qdot 800-labeled CMCs were injected on the site of injury. Transplanted cells showed to localize in the interstitial tissue among muscle fibres and to survive in vivo for up to two weeks. No signs of tumorigenesis were detected at all time points.

**Fig. 7:**
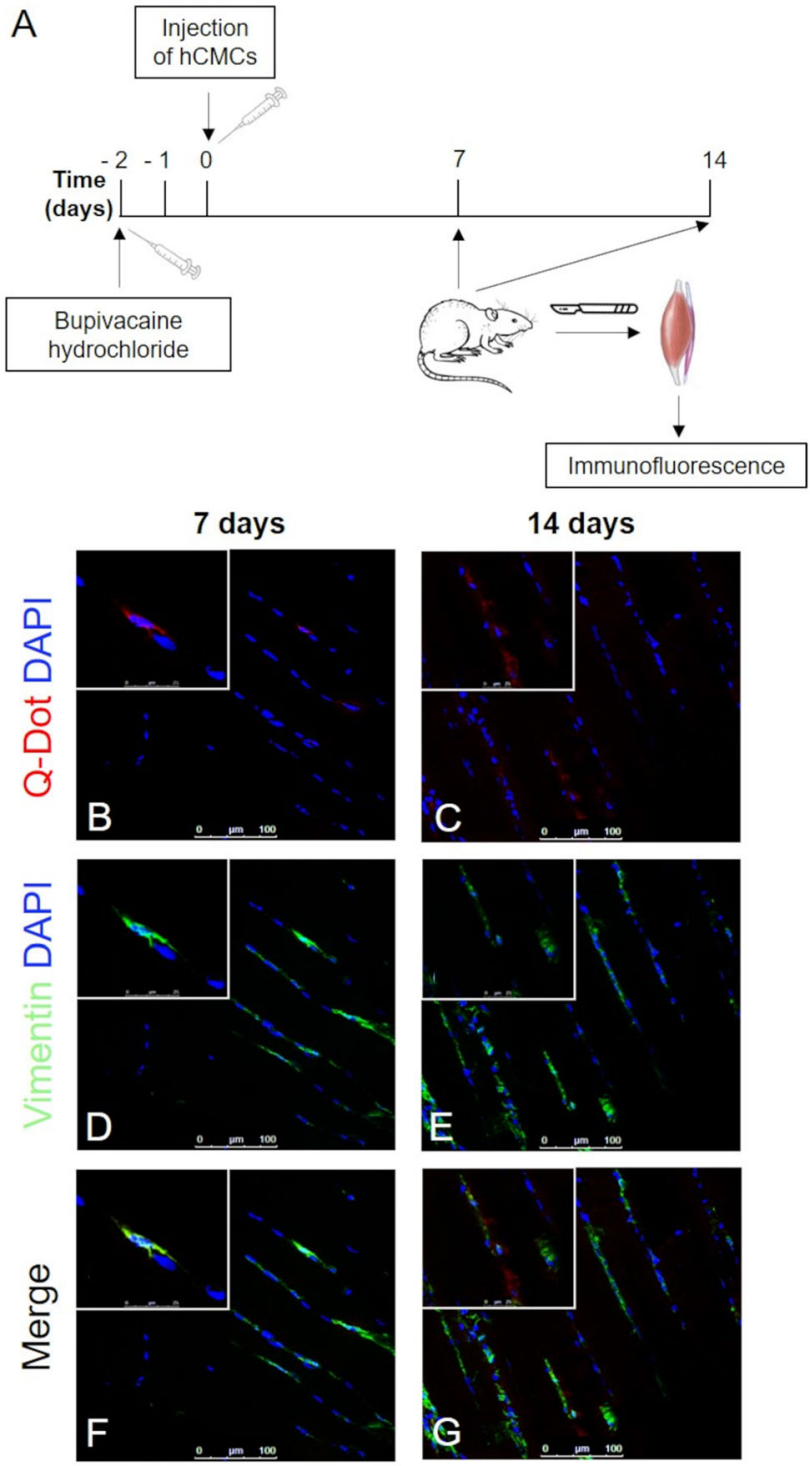
**A** *In vivo* experimental plan. **B–G** Immunofluorescence of vimentin in bupivacaine-damaged *tibialis anterior* muscle after 7 (**B**, **D**, **F**) and 14 (**C**, **E**, **G**) days from injection of Q Dot labeled CMCs. Scale bar: **B**–**G** 100 µm; **B–G** 25 µm

At 7 days from injection (Fig. 7), several CMCs (*red*) co-localized with vimentin (*green*) and aligned themselves alongside the host muscle fibers. Close to the injection site, cells showed a rounded shape probably due to a metabolic stress, whereas CMCs near to muscle cells displayed a regular and elongated morphology. At 14 days, their detected fusion with existing myofibers led to hypothesize CMCs could participate to *in vivo* muscle regeneration as satellite-like cells [18].

## Discussion

In the last decades, the identification of circulating multipotent stem cells has aroused particular interest in regenerative medicine, as human peripheral blood turns out to be an ideal cell source, characterized by easy availability and non-invasive extraction technique, as well as the possibility to isolate cell populations for autologous transplantation. Thus, the presence of circulating stem cells or multipotent progenitors can represent a key factor in promoting tissue regeneration [19, 20]. For instance, hematopoietic stem cells (HSCs) continuously circulate at very low levels in the blood to maintain a pool of hematopoietic components in the medullary compartment of peripheral bones. These HSCs can be mobilized into the blood through pharmacological treatments (administration of Granulocyte Colony-Stimulating Factor, G-CSF) or following an induced damage to the bone marrow [19, 21]. However, there is increasing evidence that not only HSCs but also other types of stem cells [i.e., mesenchymal stem cells (MSCs), endothelial progenitor cells (EPCs), and very small embryonic like stem cells (VSELs)] circulate at low level into peripheral blood under steady state conditions. Remarkably, the number of these cells seems to increase under stress conditions related to infections, inflammation, organ injury, as well as after strenuous exercises [22].

Recent investigations have also shown that the preparation of blood components such as platelet-rich products can result in increased concentrations of circulating multipotent stem cells, facilitating the isolation procedure [11, 23].

Herein, CMCs, isolated from human peripheral blood, have shown a stemness profile and differentiation capability, suggesting a regenerative potential to repair *in vivo* tissue damages. During the long-term *ex vivo* expansion, CMCs maintained a constant proliferation rate up to the 31^st^ passage in culture. Remarkably, CMC populations showed cell–cell contact inhibition, which is known to occur when adherent, non-transformed cells stop proliferating at a critical cell density forming a confluent monolayer and differentiating in a non-specific way. In line with Dominici et al. [12], immunophenotypic characterization studies highlighted the negativity to hematopoietic markers (i.e., CD14 and CD45), as well as the positivity for stem cell markers such as CD73, CD105 and CD90. Similar to multipotent cells isolated from the perivascular niche [24], they were positive for CD34, as well as for markers typically expressed by pericytes [25], such as CD13 and NG2. Furthermore, the absence of MHC class II molecules (HLA-DR) expression suggests that CMCs transplantation may be connected to a low risk of immune response. A significant experimental evidence supporting the stem nature of CMC populations is the expression of genes typically associated with self-renewal and pluripotency of embryonic/pluripotent stem cells. To corroborate gene expression results, we refer to the existing literature about the expression of pluripotency markers by mesenchymal stem cells at the undifferentiated state, finding out that CMCs present even higher levels of NANOG, OCT4 and SOX2 mRNAs in comparison with data previously reported about bone marrow-derived MSCs [26].

During neuronal differentiation, gradual changes in cell morphology after 7 days of induction with EGF and bFGF suggested that cells were entering into a commitment state. Our findings agree to several evidence demonstrating the responsiveness of many precursor cells of neural origin to EGF and bFGF [27]. After initial priming, CMCs were induced with RA and NGF, well-known factors involved in the process of neuritogenesis. RA, a developmentally regulated morphogen, has been demonstrated to control generation of primary neurons in Xenopus [28] and motor neuron differentiation [29]. Thus, RA has been widely used for embryonic stem cells since the pioneering studies of Strickland [30]. In parallel, gene expression analysis of early and late neuronal markers confirms the morphological data. Brain derived neurotrophic factor plays a critical role in the regulation of structural, synaptic and morphological plasticity. It is also involved in nerve regeneration and maintains the structural integrity and neuronal plasticity in the adult brain, regulating the synthesis of neurotransmitters [31]. Nestin, a type VI intermediate filament protein, is expressed in dividing cells during the early stages of development of the nervous tissue, where it is progressively replaced by tissue-specific intermediate filament proteins. It is also considered a marker of cell plasticity as it is expressed by multipotent stem cells [32]. Thus, the detection, even in the undifferentiated state, of nestin, BDNF and NGF suggests a differentiative potential of CMCs towards neuronal lineage. After neurogenic induction, these markers continued to be expressed until achievement of a mature differentiated state. Among early neuronal makers, GDNF, a dopaminergic neurotrophic factor secreted by glial cells, promotes the survival and differentiation of different types of neurons of the nervous system [33]. Its disappearance through the differentiation process confirms that the inductive protocol was neural lineage specific.

Regarding late neuronal markers, their expression was detected at both the mRNA and protein level at the final stages of the differentiation process. Among them, TBB3 was found to be a constitutive element of microtubules in neurons of fetal and postnatal age [34]. On the other hand, NEFM is exclusively expressed in neurons as one of the neurofilament subunit proteins which acts with microtubules and microfilaments to form and maintain the axonal structural integrity and neuronal shape, defining a very stable tubular system of the neuronal cytoskeleton [35]. Another common neuronal biomarker is NeuN, which was found to be distributed in the nuclei of mature neurons in nearly all parts of the vertebrate nervous system. Being stably expressed at developmental stages that correspond to the withdrawal of neurons from cell cycle, and/or to the initiation of terminal differentiation, NeuN is accordingly used for determination of neuronal phenotype [36]. Expressed by CMCs after 14 days of induction treatment, GLAST is a protein of the inner mitochondrial membrane that mediates the transport of L-glutamate and L- and D-aspartate. In the central nervous system, L-glutamate is the main transmitter for most of excitatory neurons, which are involved in complex physiological processes such as learning and memory [37]. Similar to TBB3, SYP and NEUROG1 were found to be expressed only by CMCs differentiated for 14 days. Synaptophysin is an integral membrane glycoprotein of presynaptic vesicles, is expressed by neuroendocrine cells and almost all the neurons of the brain and spinal cord that participate in the synaptic transmission. In parallel, the basic helix- loop- helix (bHLH) transcription factor NEUROG1 was found to be involved in the regulation of neural differentiation, confirming data published in literature [38].

As highlighted by western blot analysis, CMCs regulated the expression of NSE in response to neurogenic stimuli. The expression of this marker is a late event in neural differentiation, representing a useful index of neural maturation [39]. Neuron specific enolase is the neuronal form of the glycolytic enzyme enolase. It is found almost exclusively in neurons and cells of neuroendocrine origin and is known to influence neurotrophic activity and regulate growth, differentiation, survival and regeneration of neurons via the activation of PI3K/Akt pathway [40].

Besides NSE, Musashi and NCAM protein expression by CMCs was activated by the induction treatment. The transcription factor Musashi regulates the translation of target mRNAs to maintain neural progenitors in their proliferative state, as well as control the commitment to differentiation during neural development [41]. Neural cell adhesion molecule of the immunoglobulin superfamily engages in multiple neuronal interactions that modulate cell migration, axonal and dendritic projection, and synaptic targeting. Their downstream signal transduction events specify whether a cell moves or projects axons and dendrites to targets in the brain [42]. Thus, NCAM expression by CMCs may suggest *in vivo* migratory capacities.

The intracellular free calcium concentration plays complex signaling roles in brain, such as regulation of neuronal plasticity and neuronal survival. Homo- and heterocellular control of Ca^2+^ homeostasis supports brain physiology maintaining neural integrity. Ca^2+^ fluxes across the plasma membrane and between intracellular organelles and compartments integrate several cellular functions [43]. In this work, results of calcium flux study suggest that the synergistic action of RA and NGF was able to promote CMCs differentiation towards a functional phenotype.

Collected evidence about myogenic induction suggested that CMCs were responsive to IGF-I and ascorbic acid. It is well known that IGF-I plays several important roles during myogenesis by stimulating both cell growth and differentiation. Transgenic mice over-expressing IGF-I in skeletal muscles are characterized by hypertrophy and an enhanced muscle regeneration via activation of muscle satellite cells [44]. Furthermore, the insulin-like growth factors, IGF-I and IGF-II, are known to promote muscle differentiation in cell culture [45], and, acting through the IGF-I receptor, they have been involved in the *in vivo* formation, maintenance, and regeneration of skeletal muscle [46]. The stimulatory effect of various nutrients, especially ascorbic acid, on the extracellular matrix (ECM) production has been extensively investigated *in vitro*. Ascorbic acid plays a key role as a cofactor in the post-translational modification of collagen and increases collagen production [47]. Furthermore, it stimulates the *in vitro* proliferation of various cell types, including osteoblasts, adipocytes, chondrocytes, and odontoblasts [48]. Herein and as already reported [45], IGF-I and ascorbic acid acted synergistically on CMCs leading to morphological changes and syncytium formation. Remarkably, in response to this the induction treatment, CMCs exhibited the capacity not only to regulate gene/protein expression of key myogenic markers, but also to reduce the expression of stem cell markers (i.e., CD73 and CD105), thus confirming they have entered into the differentiation pathway.

Skeletal myogenesis is a developmental cascade involving the regulatory MYOD gene family that determines the progress of multipotential progenitors to myogenic lineage. The MYOD family is one of the basic helix loop helix transcription factors that directly modulate myocyte cell specification and differentiation [49]. In all the anatomical sites where skeletal muscle forms, commitment and terminal differentiation of skeletal muscle cells are governed by a network of four muscle-specific regulatory factor (MRFs): MYF5, muscle-specific regulatory factor 4 (MRF4; also known as MYF6), MYOD, and myogenin. MRFs activate many downstream genes to initiate muscle cell differentiation. MYOD and MYF5 constitute a cross-regulatory transcriptional network that is at the core of muscle cell determination and differentiation; disruption of this network completely abrogates skeletal muscle formation. MYF5 and MYOD are generally thought to act as determination genes, whereas myogenin is essential for the terminal differentiation of committed myoblasts [50]. During the *in vivo* myogenic differentiation, MYF5 and MyoD exhibit opposite cell-cycle fluctuations at the proliferative stage of growing myoblasts. In the differentiation phase MYF5 expression is lost, whereas MyoD continues to be expressed in the myocytes. In the final phase of maturation, MyoD is replaced by MYOG [51].

Finally, evidence derived from the *in vivo* implantation of CMCs into the damaged muscle of bupivacaine-treated rats agree to Shabbir et al. [52], who reported strings of peripheral MSC nuclei located along the length of pre-existing fibers. Additionally, neither tumour mass formation nor severe fibrosis were observed in the site of cell injection, suggesting no tumorigenic transformation of CMCs.

In this work, CMCs was isolated from peripheral blood and characterized showing a non-hematopoietic mesenchymal immunophenotype, a stable karyotype over long term culture, and multi-differentiative potential, being highly responsive to external stimuli. Interestingly, undifferentiated CMCs expressed some markers of both neuronal and myogenic lineages. However, terminal differentiation signs, at both morphological and gene expression levels, appeared only when CMCs were cultured with specific differentiation media. RA and NGF acted synergistically to induce the expression of several neuronal markers and the achievement of functional neuronal phenotype, as highlighted by the calcium flux activity and noradrenaline release. Furthermore, the temporal expression pattern of myogenic determination factors was successfully simulated *in vitro* due to the combined induction of IGF-I and ascorbic acid. Finally, *in vivo* CMCs, injected in a previously damaged skeletal muscle, integrated well within muscle fibers without any signs of tumorigenesis.

Collectively, our results suggest that CMCs can represent a valuable *in vitro* model for neurogenic and myogenic differentiation studies and drug screening. Moreover, the *in vivo* preliminary evidence indicates the potential to exploit CMCs plasticity for the therapy of degenerative diseases. However, it remains yet to ascertain whether the single cells derived from adult tissues, like peripheral blood, could differentiate into functional multiple lineages, even sustaining an *in vivo* functional engraftment. Nonetheless, future effort will be focused on investigation of tissue-specific and injury-related signals that recruit, stimulate and regulate plasticity of CMCs in animal models of degenerative diseases.
